# Physical exercise ameliorates the reduction of neural stem cell, cell proliferation and neuroblast differentiation in senescent mice induced by D-galactose

**DOI:** 10.1186/s12868-014-0116-4

**Published:** 2014-10-31

**Authors:** Sung Min Nam, Jong Whi Kim, Dae Young Yoo, Hee Sun Yim, Dae Won Kim, Jung Hoon Choi, Woosuk Kim, Hyo Young Jung, Moo-Ho Won, In Koo Hwang, Je Kyung Seong, Yeo Sung Yoon

**Affiliations:** Department of Anatomy and Cell Biology, College of Veterinary Medicine, and Research Institute for Veterinary Science, Seoul National University, Seoul, 151-742 South Korea; Department of Biochemistry and Molecular Biology, Research Institute of Oral Sciences, College of Dentistry, Gangneung-Wonju National University, Gangneung, South Korea; Department of Anatomy, College of Veterinary Medicine, Kangwon National University, Chuncheon, South Korea; Department of Neurobiology, School of Medicine, Kangwon National University, Chuncheon, South Korea

**Keywords:** D-galactose, Treadmill exercise, Hippocampus, Adult neurogenesis, Phosphorylated cAMP-response element binding protein, Brain derived neurotrophic factor, Mice

## Abstract

**Background:**

Aging negatively affects adult hippocampal neurogenesis, and exercise attenuates the age-related reduction in adult hippocampal neurogenesis. In the present study, we used senescent mice induced by D-galactose to examine neural stem cells, cell proliferation, and neuronal differentiation with or without exercise treatment. D-galactose (100 mg/kg) was injected to six-week-old C57BL/6 J mice for 6 weeks to induce the senescent model. During these periods, the animals were placed on a treadmill and acclimated to exercise for 1 week. Then treadmill running was conducted for 1 h/day for 5 consecutive days at 10-12 m/min for 5 weeks.

**Results:**

Body weight and food intake did not change significantly after D-galactose administration with/without treadmill exercise, although body weight and food intake was highest after treadmill exercise in adult animals and lowest after treadmill exercise in D-galactose-induced senescent model animals. D-galactose treatment significantly decreased the number of nestin (a neural stem cell marker), Ki67 (a cell proliferation marker), and doublecortin (DCX, a differentiating neuroblast marker) positive cells compared to those in the control group. In contrast, treadmill exercise significantly increased Ki67- and DCX-positive cell numbers in both the vehicle- and D-galactose treated groups. In addition, phosphorylated cAMP-response element binding protein (pCREB) and brain derived neurotrophic factor (BDNF) was significantly decreased in the D-galactose treated group, whereas exercise increased their expression in the subgranular zone of the dentate gyrus in both the vehicle- and D-galactose-treated groups.

**Conclusion:**

These results suggest that treadmill exercise attenuates the D-galactose-induced reduction in neural stem cells, cell proliferation, and neuronal differentiation by enhancing the expression of pCREB and BDNF in the dentate gyrus of the hippocampus.

**Electronic supplementary material:**

The online version of this article (doi:10.1186/s12868-014-0116-4) contains supplementary material, which is available to authorized users.

## Background

Neurogenesis in the subgranular zone of hippocampal dentate gyrus continues throughout lifetime. The rate of neurogenesis decreases with aging, and mild cognitive impairment is closely related to the reduction of neurogenesis [1–3]. Decreased neurogenesis is a well-known causative factor for the clinical signs of neurodegenerative disease, and enhancing hippocampal neurogenesis with methods like exercise, diet restriction, environment enrichment decelerates the progression of disease [4–8]. In particular, exercise is associated with a decreased risk of cognitive impairment and improved memory and learning capability in an animal model of Alzheimer’s disease and in aged mice [9,10]. Many studies show that exercise enhances memory function and hippocampal plasticity under normal and pathological conditions [11–14]. In a previous study, we showed that treadmill exercise positively affected cellular proliferation and neuroblast differentiation in the dentate gyrus of type 2 diabetic model [15].

D-galactose (D-gal) is a reducing sugar and over-dose results in the accumulation of galactitol and the formation of advanced glycation end products [16,17]. As a result, increased oxidative stress caused aging-like changes in brain; thus, D-gal has been used in chemically induced aging models [16–23]. Chronically exposing mice to systemic D-gal causes decreased adult neurogenesis in the hippocampal dentate gyrus [18,21–23]. In addition, administrating D-gal to mice impairs learning and memory by reducing expression of pre- and post-synaptic proteins [24]. D-gal-induced brain aging is partially caused by an impaired astrocyte and cholinergic system [19,20]. However, the effects of treadmill exercise in the aspect of hippocampal adult neurogenesis have not been confirmed in the D-gal-induced aging model.

As a common mediator of various signal transduction pathways, phosphorylated cAMP-response element binding protein (pCREB) binds to the cAMP-response element (CRE) in the promoter region of target genes [25]. pCREB has been highlighted for its role in adult neurogenesis which has been demonstrated *in vitro* and *in vivo* studies [26–29]. During adult neurogenesis, pCREB expression site is localized at the subgranular zone of hippocampal dentate gyrus and pCREB expression period overlaps with doublecortin (DCX) expression [30,31]. But until now, the role of pCREB during adult neurogenesis after treadmill exercise in the D-gal-induced aging model is not clear. Therefore, we investigated the effect of treadmill exercise on hippocampal neurogenesis and pCREB expression in the hippocampus of the D-gal-induced aging model with or without exercise.

## Methods

### Experimental animals

Five-week-old male C57BL/6 J mice were purchased from Japan SLC, Inc. (Shizuoka, Japan). The animals were housed under conventional conditions with adequate temperature (23°C) and humidity (60%) control on a 12-h light-dark cycle. Food and water were available *ad libitum*. The procedures for handling and caring of animals followed the Guide for the Care and Use of Laboratory Animals issued by Institute of Laboratory Animal Resources, USA, 1996, and the experimental protocol was approved by the Institutional Animal Care and Use Committee (IACUC) of Seoul National University (approval no. SNU-120305-5). All experiments were conducted in a manner to minimize the number of animals used and the suffering caused by the procedures used in this study.

### Drug and exercise treatment

Following a 1 week acclimation to laboratory conditions, the animals were divided into four groups (*n* =13 in each group): sedentary vehicle-treated (S-Veh), exercise vehicle-treated (Ex-Veh), sedentary D-gal-treated (S-D-gal), and exercise D-gal-treated (Ex-D-gal) groups. D-gal was subcutaneously administered (100 mg/kg/day) to 6-week-old mice once/day for 6 weeks. In addition, Ex-Veh and Ex-D-gal animals were familiarized with running on a motorized treadmill (Model 1050 Exer3/6; Columbus Instruments, Columbus, OH, USA) for 1 week at 6 weeks of age. The running speed and durations were 10 m/min, 20 min for the first day, with an increment of 10 min/day until reaching 60 min/day to fulfill the 70% of maximal oxygen consumption [32]. After becoming familiarized with the treadmill, electrical stimulation to encourage the mice to run was discontinued to avoid pain stress beginning at 7 weeks of age. The running duration was 60 min/day, and the running speed was increased gradually from 10 to 12 m/min. The speed was accelerated 1 m/min every 2 weeks.

### Check for body weight and food intake

Body weight was measured on Monday morning of every week and at the end of the experiment. Food intake was measured, and corrected for spillage by weighing the jars containing food every week between 9.00 to 10.00 h. Data are expressed as gram/day/body weight (g).

### Tissue processing

At the end of the experiment, all mice were anesthetized with mixture of zolazepam and tiletamine (30 mg/kg, Virbac, Carros, France) and perfused transcardially with 0.1 M phosphate-buffered saline (PBS, pH 7.4) followed by 4% paraformaldehyde in 0.1 M phosphate-buffer (PB, pH 7.4). The brains were removed and postfixed in the same fixative for 12 h. For brain derived neurotrophic factor (BDNF) and pCREB immunohistochemistry, brain tissues (*n* =3) were dehydrated with graded concentrations of alcohol and xylene for embedding in paraffin. Three μm-thick sections were serially cut using a microtome (Leica, Wetzlar, Germany), and they were mounted onto silane-coated slides (Muto-glass, Tokyo, Japan). For immunohistochemical staining except BDNF and pCREB, brain tissues (n =5) were cryoprotected by infiltration with 30% sucrose for 1-2 days. Following equilibration in 30% sucrose in PBS, the brain were serially cut on a freezing sliding microtome (Leica, Wetzlar, Germany) into 30-μm-thick coronal sections. The sections were collected in six-well plates containing PBS and stored in storage solution until further processing.

### Immunohistochemistry

In order to obtain accurate data, immunohistochemical staining was carefully conducted under the same conditions. For paraffin sections, five tissue sections were selected at 30 μm apart between 1.46 and 2.46 mm posterior to the bregma in reference to a mouse atlas [33] for each animal. BDNF and pCREB immunohistochemical staining was performed according to a previous study using paraffin-embedded block [34]. Briefly, the sections were placed in 400-mL jars filled with citrate buffer (pH 6.0) and heated in a microwave oven (Optiquick Compact, Moulinex) operating at a frequency of 2.45 GHz and 800 W power setting. After three heating cycles of 5 min each, slides were allowed to cool at room temperature and were washed in PBS. Free floating sections were also carefully processed under the same conditions to obtain accurate data for immunohistochemistry. Five sections in 180 μm apart from each other were selected between 1.46 mm and 2.46 mm posterior to the bregma for each animal with reference to a mouse atlas [33].

The sections were sequentially treated with 0.3% hydrogen peroxide (H_2_O_2_) in 0.1 M PBS and 10% normal goat or rabbit serum in 0.1 M PBS. Then, they were incubated with diluted chicken anti-nestin antibody (1:250; Novus, Littleton, CO, USA), rabbit anti-Ki67 antibody (1:1,000; Abcam, Cambridge, UK), goat anti-DCX antibody (1:50; Santa Cruz Biotechnology, Santa Cruz, CA, USA), rabbit anti-BDNF (1:500; Novus, Littleton, CO, USA) or rabbit anti-pCREB (1:1,000; Millipore, Temecula, CA, USA) overnight, and subsequently exposed to biotinylated goat anti-chicken, rabbit anti-goat, or goat anti-rabbit IgG (diluted 1:200; Vector Labs., Burlingame, CA, USA) and streptavidin peroxidase complex (diluted 1:200, Vector Labs.). Then, the sections were visualized by reaction with 3,3’-diaminobenzidine tetrahydrochloride (Sigma, St. Louis, MO, USA).

To elucidate the effects of exercise in this experiment, the corresponding areas of the dentate gyrus were measured from five sections per animal. The number of Ki67-, DCX-, and pCREB-positive cells in all groups was counted using an image analysis system equipped with a computer-based CCD camera (Optimas 6.5 software, CyberMetrics, Scottsdale, AZ, USA). Additionally, total dendritic length of DCX-positive neuroblasts (15 DCX-positive neuroblasts in each mouse) was measured using NIH Image 1.59 software (ImageJ) with NeuronJ plug-in [35,36].

In addition, the region of interest in the dentate gyrus was analyzed using an image analysis system. Images were calibrated into an array of 512 × 512 pixels corresponding to a total dentate gyrus (100× primary magnification). Pixel resolution was 256 gray levels. The intensity of nestin, DCX and BDNF immunoreactivity was evaluated by means of a relative optical density (ROD), which was obtained after transforming the mean gray level using the formula: ROD = log (256/mean gray level). ROD of the background was determined in unlabeled portions using ImageJ and the value was subtracted for correction, yielding high ROD values in the presence of preserved structures and low. The ROD ratio of was calibrated as a percentage.

### Western blot

To confirm the effects of D-gal and/or exercise on nestin, DCX and BDNF protein levels, mice (*n* =5 in each group) were sacrificed by decapitation after deep anesthesia. Brains were rapidly removed and hippocampi were dissected out with a surgical blade. Hippocampi were immediately frozen in liquid nitrogen and stored at -80°C until further processing. Tissues were homogenized in 50 mM PBS (pH 7.4) containing 0.1 mM ethylene glycol bis (2-aminoethyl Ether)-*N*,*N*,*N*′,*N′* tetraacetic acid (EGTA) (pH 8.0), 0.2% Nonidet P-40, 10 mM ethylendiamine-tetraacetic acid (EDTA) (pH 8.0), 15 mM sodium pyrophosphate, 100 mM β-glycerophosphate, 50 mM NaF, 150 mM NaCl, 2 mM sodium orthovanadate, 1 mM phenylmethylsulfonyl fluoride (PMSF) and 1 mM dithiothreitol (DTT). After centrifugation, the protein level was determined in the supernatants using a Micro BCA protein assay kit using bovine serum albumin as the standard (Pierce Chemical, Rockford, IL). Aliquots containing 50 μg of total protein were boiled in a loading buffer that contained 150 mM Tris (pH 6.8), 3 mM DTT, 6% SDS, 0.3% bromophenol blue and 30% glycerol. The aliquots were then loaded onto a polyacrylamide gel. After electrophoresis, the proteins were transferred from the gel to nitrocellulose transfer membranes (Pall Corp., East Hills, NY). To reduce background staining, the membranes were incubated with 5% non-fat dry milk in PBS containing 0.1% Tween 20 for 45 min, followed by incubation with chicken anti-nestin antibody (1:10,000), goat anti-DCX antibody (1:1,000), or rabbit anti-BDNF (1:1,000), peroxidase-conjugated anti-chicken, anti-goat or anti-rabbit IgG and an enhanced luminol-based chemiluminescent (ECL) kit (Pierce Chemical). The blot was densitometrically scanned for the quantification of ROD of each band using NIH Image 1.59 software.

### Statistical analysis

Data represent the means of experiments performed for each experimental area. Differences among the means were statistically analyzed by two-way analysis of variance followed by Bonferroni’s post-tests using GraphPad Prism 5.0 software (GraphPad Software Inc., La Jolla, CA, USA) in order to elucidate differences between D-gal × exercise. A p <0.05 was considered significant.

## Results

### Effects of exercise on body weight and food intake in adult and D-gal treated mice

**Figure 1:**
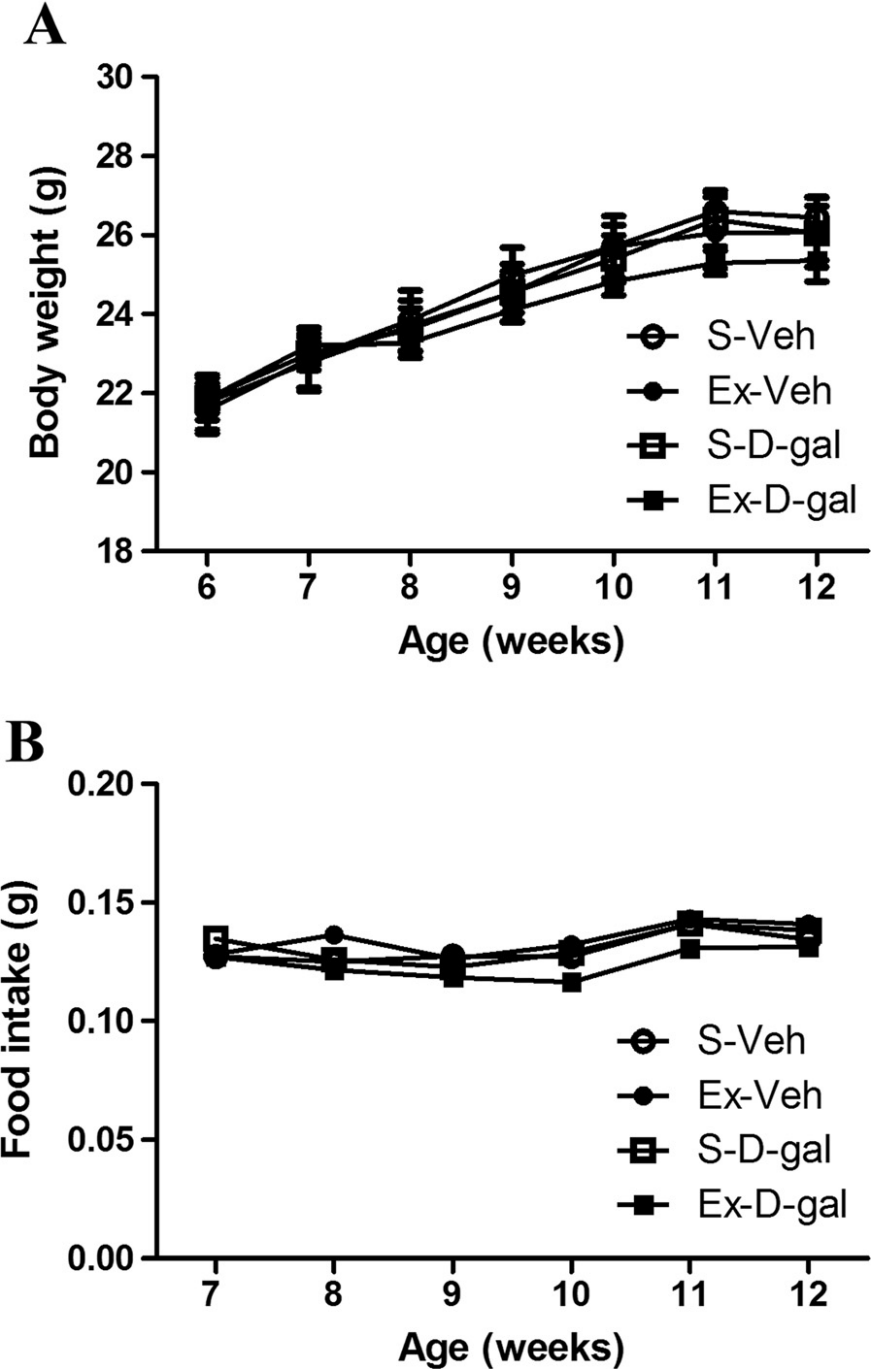
**Effects of exercise on body weight and food intake in adult and D-gal treated mice.** Changes in body weight **(A)** and food intake **(B)** of sedentary-vehicle (S-Veh), exercised-vehicle (Ex-Veh), sedentary-D-galactose (S-D-gal), and exercised- D-gal (Ex-D-gal) groups (All data are shown as the mean ± standard errors for mean (SEM).

Body weight was increased with age. Body weight and food intake was higher in Ex-Veh group compared with those in S-Veh, S-D-gal or Ex-D-gal groups, but significant differences were not detected. Body weight and food intake in Ex-D-gal group, was slightly low compared to that in the S-D-gal group (*P* >0.05) (Figure 1).

### Effects of exercise on neural stem cells in adult and D-gal treated mice

**Figure 2:**
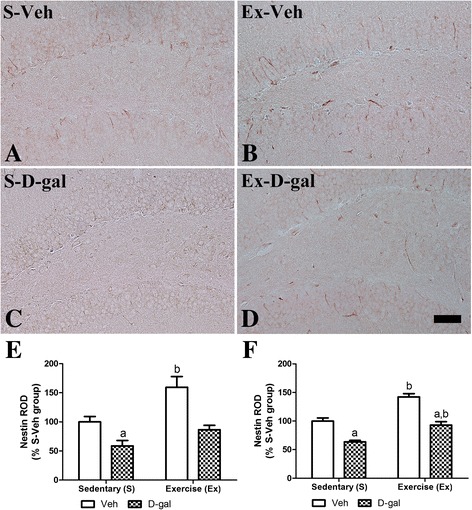
**Effects of exercise on neural stem cells in adult and D-gal treated mice.** Immunohistochemistry for nestin in the dentate gyrus of the S-Veh **(A)**, Ex-Veh **(B)**, S-D-gal **(C)**, and Ex-D-gal **(D)** groups. Granule cell layer, GCL; polymorphic layer, PoL; ML, molecular layer. Scale bar =50 μm. **E**: Relative optical density (ROD) demonstrated as percentages of S-Veh in nestin immunoreactivity per section in all groups. **F**: ROD of the immunoblot bands is demonstrated as percent values (^a^ *P* <0.05, between vehicle vs. D-gal groups, ^b^ *P* <0.05, between sedentary vs. exercise groups). All data are shown as the mean ± SEM.

In the S-Veh group, the nestin immunoreactive cells and fibers were detected in the subgranular zone and granule cell layer of dentate gyrus (Figure 2A). In the Ex-Veh group, nestin-immunoreactive cells and fibers were prominent compared to that in the S-Veh group (Figure 2B). In this group, nestin immunoreactivity increased significantly to 159.4% of that in the S-Veh group (*P* <0.01) (Figure 2F and Additional file 1: Figure S1). In addition, nestin protein levels were significantly increased to 142.2% of that in the S-Veh group (*P* <0.01). In the S-D-gal group, few nestin immunoreactive cells and fibers were detected in the dentate gyrus (Figure 2C). In this group, nestin immunoreactivity decreased to 58.7% of that in the S-Veh group (*P* >0.05) (Figure 2E). Additionally, nestin protein levels in the hippocampal homogenates of the S-D-gal group was significantly lowered to 63.7% of that in the S-Veh group (*P* <0.01) (Figure 2F and Additional file 1: Figure S1). In the Ex-D-gal group, nestin immunoreactive cells and fibers increased compared to of S-D-gal group (*P* >0.05) (Figure 2D). In this group, nestin immunoreactivity increased significantly to 156.2% of that in the S-D-gal group, however, its level was significantly lowered as 54.3% of that in the Ex-Veh group (*P* <0.01) (Figure 2E). In the Ex-D-gal group, nestin protein levels were significantly increased to 145.9% of that in the S-D-gal group (*P* <0.01) (Figure 2F and Additional file 1: Figure S1). Additionally, in this group, nestin protein levels were significantly reduced by D-gal treatment compared to the Ex-Veh group (65.3% of the Ex-Veh group) (*P* <0.01) (Figure 2F).

### Effects of exercise on cell proliferation in adult and D-gal treated mice

**Figure 3:**
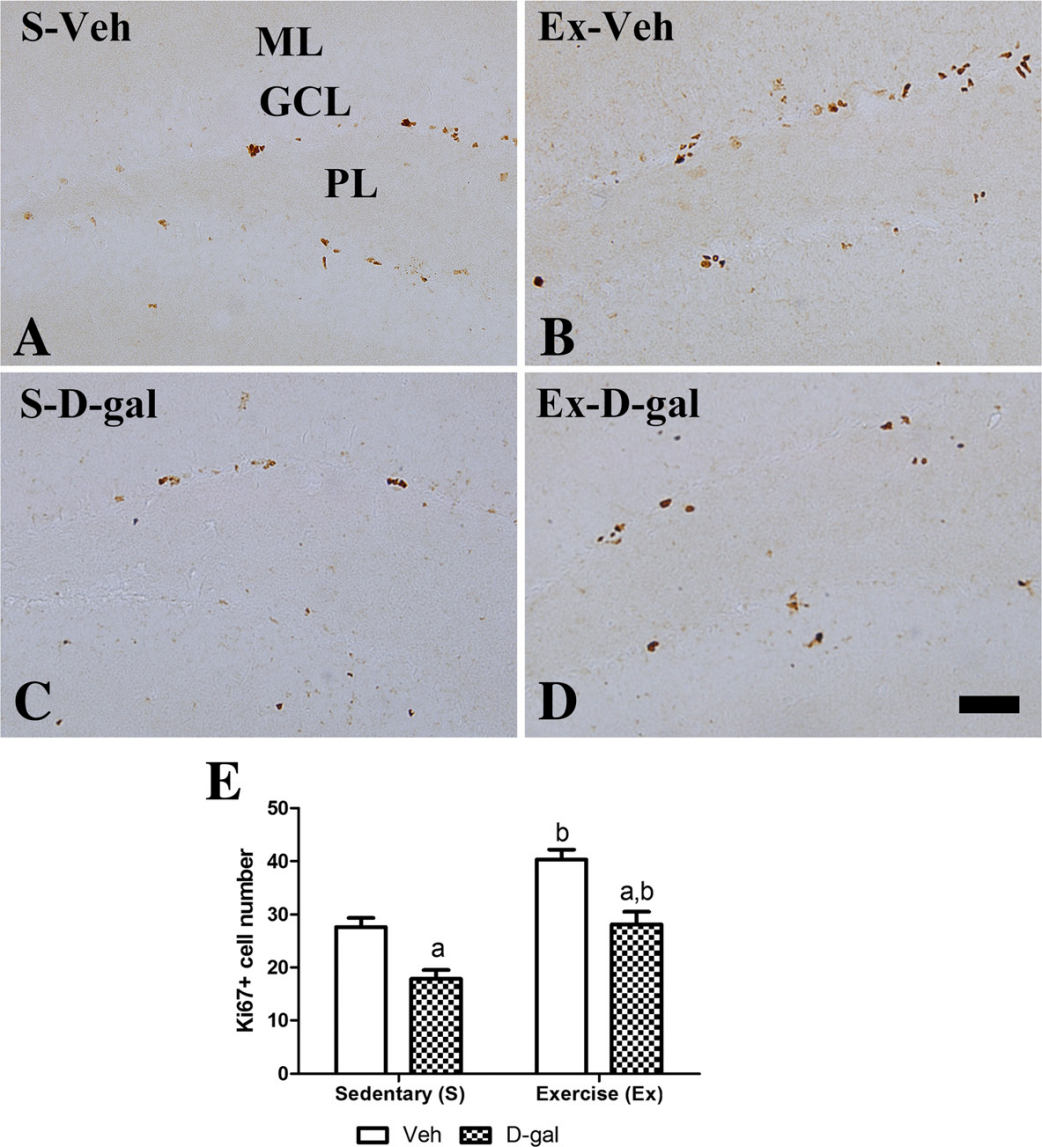
**Effects of exercise on cell proliferation in adult and D-gal treated mice.** Immunohistochemistry for Ki67 in the dentate gyrus of the S-Veh **(A)**, Ex-Veh **(B)**, S-D-gal **(C)**, and Ex-D-gal **(D)** groups. Ki67-positive nuclei are observed in the subgranular zone of the dentate gyrus. Granule cell layer, GCL; polymorphic layer, PoL; ML, molecular layer. Scale bar =50 μm. **E**: Mean number of Ki67-positive nuclei per section in all groups (^a^ *P* <0.05, between vehicle vs. D-gal groups, ^b^ *P* <0.05, between sedentary vs. exercise groups). All data are shown as the mean ± SEM.

In the S-Veh group, Ki67-positive nuclei were clustered mainly in the subgranular zone of the dentate gyrus (Figure 3A). In this group, the average number of Ki67-immunoreactive nuclei was 29.2 per section (Figure 3E). In the Ex-Veh group, the average number of Ki67-positive nuclei increased significantly compared to that in the S-Veh group and was 41.0 per section (*P* <0.01) (Figure 3B and E). In the S-D-gal group, the average number of Ki67-positive cells was lowest with 19.0 per section (Figure 3C and E). In the Ex-D-gal group, the number of Ki67-positive cells increased significantly compared to that in the S-D-gal group (*P* <0.01) and was similar to that in the S-Veh group (Figure 3D and E). However, the increase in average number of Ki67-positive cells was significantly low in the Ex-D-gal group compared to the Ex-Veh group (*P* <0.01) (Figure 3E).

### Effects of exercise on neuroblast differentiation in adult and D-gal treated mice

**Figure 4:**
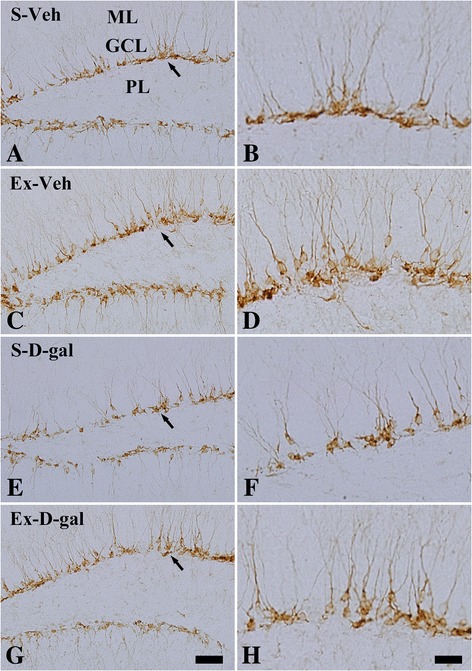
**Effects of exercise on neuroblast differentiation in adult and D-gal treated mice.** Immunohistochemistry for doublecortin (DCX) in the dentate gyrus of the S-Veh **(A and B)**, Ex-Veh **(C and D)**, S-D-gal **(E and F)**, and Ex-D-gal **(G and H)** groups. Granule cell layer, GCL; polymorphic layer, PoL; ML, molecular layer. Scale bar =50 μm **(A, C, E, and G)**, 25 μm **(B, D, F, and H)**.

**Figure 5:**
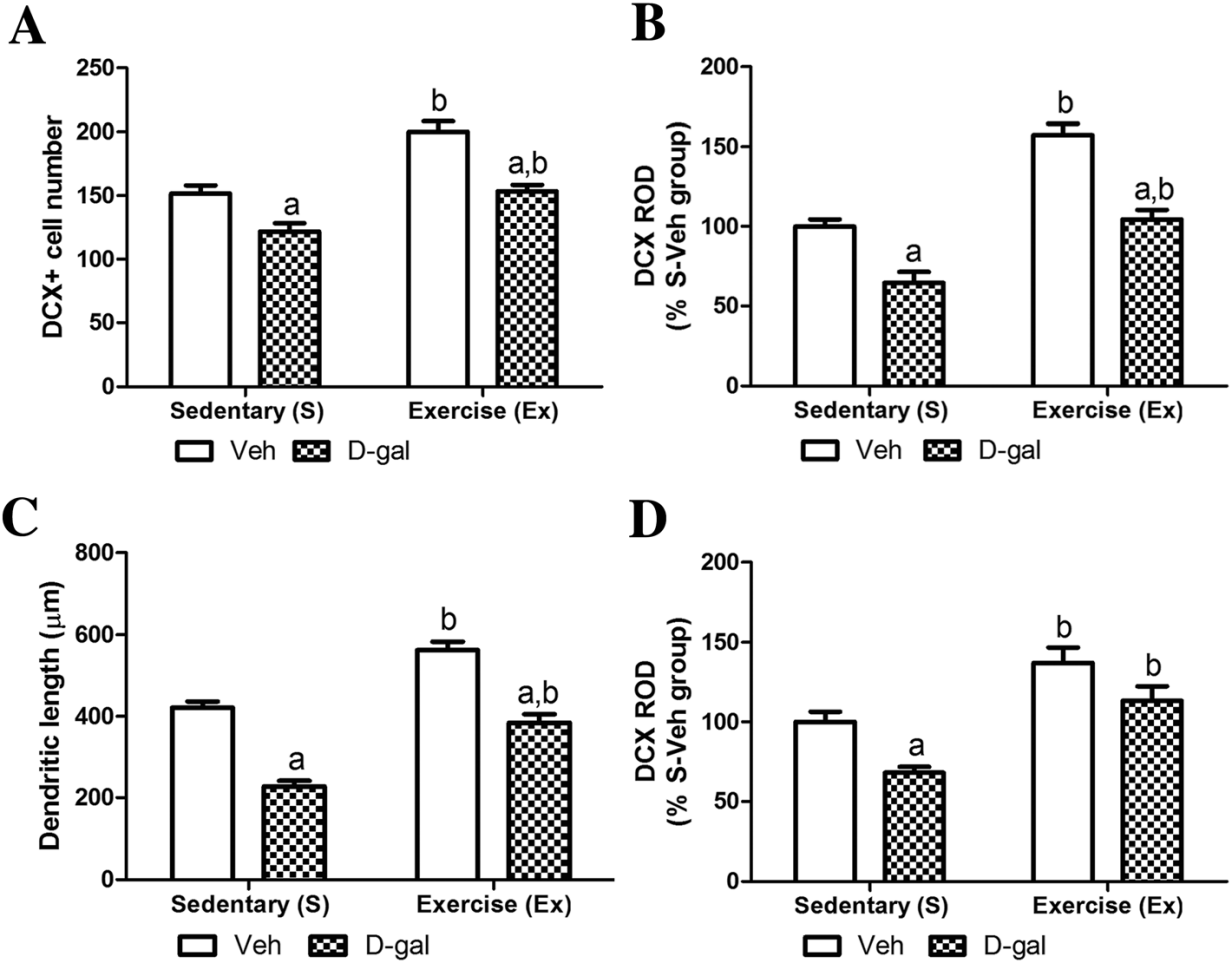
**Mean number, relative optical density, dendritic length of DCX-immunoreactive neuroblasts and its protein level.** Mean number of DCX-immunoreactive neuroblasts per section **(A)** and relative optical density (ROD) demonstrated as percentages of S-Veh in DCX immunoreactivity per section **(B)** in the dentate gyrus of S-Veh, Ex-Veh, S-D-gal, and Ex-D-gal groups. **C**: Mean of dendritic length of DCX-immunoreactive neuroblasts in the S-Veh, Ex-Veh, S-D-gal, and Ex-D-gal groups. **D**: ROD of the immunoblot bands is demonstrated as percent values (^a^ *P* <0.05, between vehicle vs. D-gal groups, ^b^ *P* <0.05, between sedentary vs. exercise groups). All data are shown as the mean ± SEM.

In S-Veh group, DCX-immunoreactive neuroblasts were observed in the subgranular zone of dentate gyrus, and they had dendrites extending into the molecular layer of the dentate gyrus (Figure 4A and B). In this group, the average number of DCX-immunoreactive neuroblasts in this group was 144.0 (Figure 5A). The average dendritic length of DCX-immunoreactive neuroblasts in this group was 421.0 μm (Figure 5C).

In the Ex-Veh group, DCX-immunoreactive neuroblasts have prominently developed dendrites (Figure 4C and D) and the number of DCX-immunoreactive neuroblasts increased significantly compared to that in the S-Veh group with 194.3 per section (*P* <0.01) (Figure 5A). In addition, DCX immunoreactivity increased significantly to 157.3% of that in the S-Veh group (P <0.01) (Figure 5B). The average length of dendrites was significantly increased to 562.5 μm (*P* <0.01) and the complexity of the dendritic branching was enhanced in the Ex-Veh group (Figure 5C). DCX protein levels in Ex-Veh group were significantly increased to 137.1% of that in the S-Veh group (*P* <0.01) (Figure 5D and Additional file 1: Figure S1).

In the S-D-gal group, DCX-immunoreactive neuroblasts and their dendrites decreased significantly compared to those in the S-Veh group (*P* <0.05) (Figure 4E and F). In this group, the average number of DCX-immunoreactive neuroblasts was the lowest among groups at 111.8 per section (Figure 5A). In addition, DCX immunoreactivity decreased significantly with 64.5% of that in the S-Veh group (*P* <0.01) (Figure 5B). The average length of the dendrite was also decreased to 227.5 μm and the complexity of dendritic branching was decreased (Figure 5C). The DCX protein levels in the hippocampal homogenates of the S-D-gal group were significantly lowered to 68.0% of that in the S-Veh group (*P* <0.05) (Figure 5D and Additional file 1: Figure S1).

In the Ex-D-gal group, DCX-immunoreactive neuroblasts significantly increased (*P* <0.01), and their dendrites were well-developed compared to those in the S-D-gal group (Figure 4G, H, Figure 5A and B). The number of DCX-immunoreactive neuroblasts and DCX immunoreactivity in this group was similar to that in the S-Veh group (Figure 5A and B). In the Ex-D-gal group, average dendritic length of DCX immunoreactive neuroblasts increased as 384.30 μm, representing 91.3% and 168.9% of the S-Veh and S-D-gal group, respectively (Figure 5C). Additionally, in Ex-D-gal group, the average number, dendritic length of DCX-immunoreactive neuroblasts and DCX immunoreactivity was significantly decreased compared to the Ex-Veh group (*P* <0.01) (Figure 5A, B and C). DCX protein levels were significantly increased to 166.6% of that in the S-D-gal groups (*P* <0.01) (Figure 5D and Additional file 1: Figure S1). However, compared to the Ex-Veh group, DCX protein levels was low in the hippocampal homogenates compared to that in the Ex-D-gal group (*P* >0.05) (Figure 5D).

### Effects of exercise on pCREB expression in adult and D-gal treated mice

**Figure 6:**
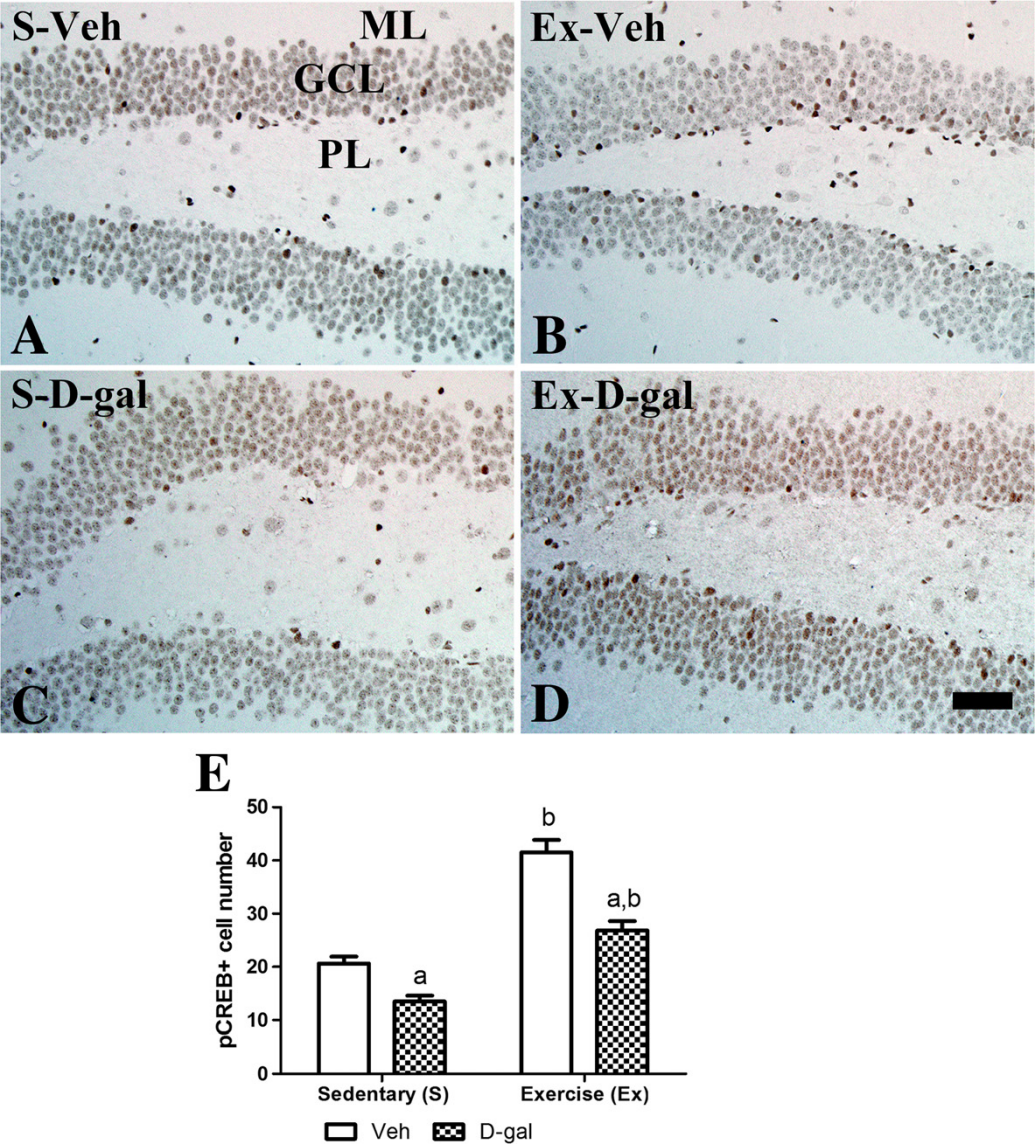
**Effects of exercise on pCREB in adult and D-gal treated mice.** Immunohistochemistry for phosphorylated cAMP-response element binding protein (pCREB) in the dentate gyrus the S-Veh **(A)**, Ex-Veh **(B)**, S-D-gal **(C)**, and Ex-D-gal **(D)** groups. Granule cell layer, GCL; polymorphic layer, PoL; ML, molecular layer. Bar =50 μm. **E**: Mean number of pCREB-positive nuclei per section in all groups ( ^a^ *P* <0.05, between vehicle vs. D-gal groups, ^b^ *P* <0.05, between sedentary vs. exercise groups). All data are shown as the mean ± SEM.

In the S-Veh group, pCREB-immunoreactive nuclei were mainly detected in the subgranular zone of the dentate gyrus (Figure 6A). In this group, the average number of pCREB-immunoreactive nuclei was 20.7 per section (Figure 6E). In the Ex-Veh group, the average number of pCREB-immunoreactive nuclei increased significantly compared to that in the S-Veh group and was 41.5 per section (*P* <0.05) (Figure 6B and E). In the S-D-gal group, pCREB-immunoreactive nuclei decreased significantly compared to that in the S-Veh group and the average number of pCREB immunoreactive nuclei was 13.5 per section (*P* <0.01) (Figure 6C and E). In the Ex-D-gal group, the average number of pCREB-immunoreactive nuclei increased significantly compared to that in the S-D-gal group at 26.8 per section (*P* <0.01) (Figure 6D and E). In this group, the average number of pCREB-immunoreactive nuclei was higher than that in the S-Veh group and significantly lower than the Ex-Veh group (*P* <0.01) (Figure 6E).

### Effects of exercise on BDNF expression in adult and D-gal treated mice

**Figure 7:**
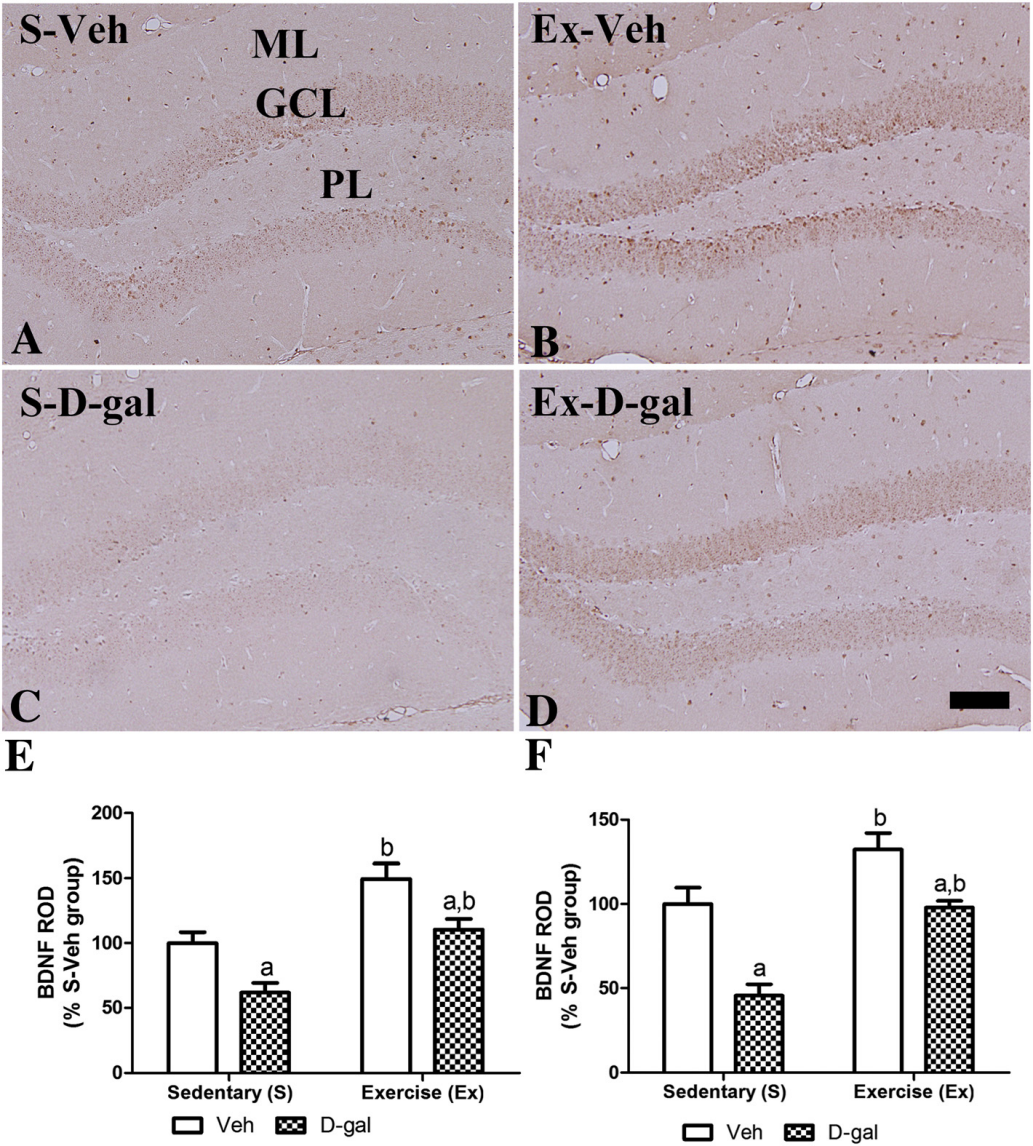
**Effects of exercise on BDNF in adult and D-gal treated mice.** Immunohistochemistry for brain-derived neurotrophic factor (BDNF) in the dentate gyrus the S-Veh **(A)**, Ex-Veh **(B)**, S-D-gal **(C)**, and Ex-D-gal **(D)** groups. Granule cell layer, GCL; polymorphic layer, PoL; ML, molecular layer. Bar =100 μm. **E**: Relative optical density (ROD) demonstrated as percentages of S-Veh in BDNF immunoreactivity per section in all groups. **F**: ROD of the immunoblot bands is demonstrated as percent values (^a^ *P* <0.05, between vehicle vs. D-gal groups, ^b^ *P* <0.05, between sedentary vs. exercise groups). All data are shown as the mean ± SEM.

In the S-Veh group, BDNF-immunoreactive cells were mainly detected in the granule cell layer of the dentate gyrus (Figure 7A). In the Ex-Veh group, the BDNF immunoreactivity was increased prominently as 149.4% of that in the S-Veh group (*P* <0.01) and the mean BDNF protein levels in hippocampus were increased as 134.3% of the S-Veh group (*P* <0.05) (Figure 7B, E, F and Additional file 1: Figure S1). In the S-D-gal group, the BDNF immunoreactivity was significantly decreased as 61.8% of that in the S-Veh group (*P* <0.05) and its protein levels showed were significantly decreased as 40.4% of the S-Veh group (*P* <0.01) (Figure 7C, E, F and Additional file 1: Figure S1). However, in the Ex-D-gal group, the BDNF immunoreactivity was increased compared to that in S-D-gal group as 178.8% of that in the S-D-gal and 110.4% of that on the S-Veh group (*P* <0.01) (Figure 7D and E). Additionally, in this group, BDNF protein levels were restored to as 103.3% of that on the S-Veh group and 167.2% of that in the S-D-gal (Figure 7F and Additional file 1: Figure S1). BDNF immunoreactivity and protein levels were low as 73.9% and 76.9% of the in Ex-Veh group (*P* <0.01) (Figure 7E and F).

## Discussion

In the present study, we observed changes in body weight and food intake of adult and D-gal-treated mice with or without physical exercise. We did not observe significant changes of body weight and food intake between groups. However, body weight and food intake tended to be higher in the Ex-Veh group and lower in the Ex-D-gal group although statistical significance was not detected.

We induced senescence in mice by administering D-gal for 6 weeks and observed neural stem cells, cell proliferation, neuroblast differentiation, and pCREB expression in the hippocampal dentate gyrus. We evaluated neural stem cell by staining nestin because most nestin-expressing neural stem cells are destined to neuronal fate [37–40]. Nestin, Ki67 and DCX immunohistochemical staining revealed that D-gal treatment reduced neural stem cells, cell proliferation and neuronal differentiation in the subgranular zone of dentate gyrus. Along with reduction of the cell number, the negative effects of D-gal on dendritic length and complexity of DCX immunoreactive neuroblasts were apparent. Protein expression level of the nestin and DCX showed similar pattern with immunohistochemistry as D-gal-induced reduction of nestin, Ki67, DCX, BDNF and pCREB proteins in the hippocampus. The results presented here coincided with previous studies that both D-gal-induced aging and natural aging reduce adult hippocampal neurogenesis [18,22,23,41]. However, there are controversial results about the neural stem cell pool. Some studies reported that natural aging animal shows a reduction of neural stem cell pool [5,6], while other studies do not observe any aging induced reduction of neural stem cell pool [42–44].

In the present study, we also investigated whether treadmill exercise attenuates the reduction in neural stem cells, cell proliferation and neuroblast differentiation induced by D-gal treatment. Enhanced neural stem cells, cell proliferation and neuroblast differentiation were found in both the Ex-Veh and Ex-D-gal groups compared with those in the S-Veh and S-D-gal groups, respectively. In addition, exercise-induced increases in the dendritic length and complexity of DCX-immunoreactive neuroblasts were confirmed and the positive effects of exercise were prominent in D-gal-treated group. Protein expression level of the nestin and DCX showed similar pattern with immunohistochemistry as exercise-induced increase of above proteins in the hippocampus. These results suggest that physical exercise significantly increases neural stem cells, cell proliferation and neuroblast differentiation in the hippocampal dentate gyrus in both adult and chemical-induced aging mice. Our present results are supported by previous studies showing that exercise increases the neurogenesis in subventricular zone and dentate gyrus of rats and mice [9,15,45,46]. In addition, exercise increased neural stem cell and it enhances neuronal proliferation and differentiation by the asymmetric cell division of neural stem cell [47,48]. However, the effects of treadmill exercise on hippocampal neuronal activity and adult neurogenesis are controversial depending on the exercise conditions such as intensity and duration period [49,50]. High intensity treadmill exercise results in slight or no significant changes in adult hippocampal neurogenesis [50]. In the present study, we used the exercise protocol that was confirmed in a previous study [15,34]. We also observed that cell proliferation and neuroblast differentiation were lower in the Ex-D-gal group than those in the Ex-Veh group. This result was supported by previous study reporting that positive effects on cell proliferation induced by treadmill exercise decreases with aging in rats [51]. Also we suggest D-gal induced reduction in nestin-expressing neural stem cells can be one of the reasons in the reduction of cell proliferation and neuronal differentiation. In the contrary, exercise induced increase in nestin-expressing neural stem cells can be related with the enhanced cell proliferation and neuronal differentiation.

We additionally investigated whether exercise treatment modulated pCREB expression in the hippocampal dentate gyrus. pCREB expression decreased in the S-D-gal group, whereas it increased in treadmill-exercised adult and D-gal-treated groups. The change of pCREB showed similar pattern with that of the nestin, Ki67 and DCX. Some studies have reported that pCREB plays roles in neurogenesis [26–29]. The genetic disruption of CREB by null mutation results in perinatal death and a conditional mutation causes progressive neurodegeneration in the hippocampus [52]. On the contrary, enhancing the pCREB pathway rescues amyloid beta-induced impairments in synaptogenesis, neurogenesis, and memory [28,53]. These results support our present results and we suggest that the change in pCREB by D-gal and exercise is closely related with the degree of hippocampal cell proliferation and neuronal differentiation.

Along with pCREB, we investigated the BDNF protein expression in the hippocampus using immnunohistochemistry and immunoblot. We observed that D-gal treatment caused significant reduction in BDNF and exercise restored the reduction of BDNF. BDNF’s positive roles in neurogenesis and synaptic plasticity corroborate BDNF relation with adult hippocampal neurogenesis in our present results [35,54–56]. Additionally, BDNF is neuroprotective against brain injury and increase of BDNF is related with enhancement in aging dependent cognitive impairment [56–58]. CREB is a downstream molecule of BDNF signaling, increase of pCREB cause target gene transcription including BDNF [59,60]. Though, we did not demonstrate the positive feedback relationship between BDNF and pCBEB, we can suggest that change of BDNF by D-gal and exercise is also closely related with adult hippocampal neurogenesis.

## Conclusion

Treadmill exercise mitigated the reduction of neural stem cells, cell proliferation, and neuroblast differentiation in a D-gal-induced senescence mouse model by enhancing the transcription factor pCREB and BDNF protein expression in the hippocampal dentate gyrus.

## Additional file

Additional file 1: Figure S1. Western blot analysis of nestin, DCX and BDNF in the hippocampus.
