# Supplementary figures and images for: Physical exercise ameliorates the reduction of neural stem cell, cell proliferation and neuroblast differentiation in senescent mice induced by D-galactose
Source: BMC Neurosci. 2014 Oct 31;15:116. doi: 10.1186/s12868-014-0116-4 (PMC4219098; doi:10.1186/s12868-014-0116-4)

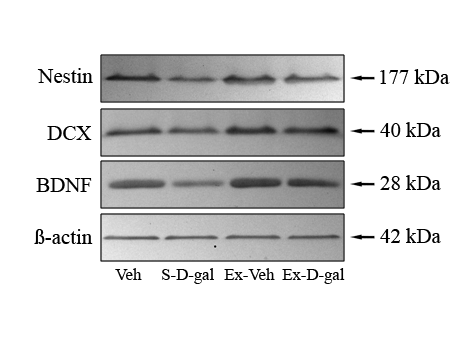

Supplement: Additional file 1: Figure S1. — Western blot analysis of nestin, DCX and BDNF in the hippocampus. [file 12868_2014_116_MOESM1_ESM.tiff]
